# Targeting neutrophil elastase is a promising direction for future cancer treatment

**DOI:** 10.1007/s12672-024-01010-3

**Published:** 2024-05-15

**Authors:** Wangqiang Jia, Yudong Mao, Qianwen Luo, Jiang Wu, Quanlin Guan

**Affiliations:** 1 The First Clinical Medical College of Lanzhou University, Lanzhou, China; 2 Department of Oncology Surgery, the First Hospital of Lanzhou University, No. 1, Donggang West Road, Lanzhou, 730000 Gansu Province China

**Keywords:** Neutrophil elastase, Cancer, Proliferation, Metastasis, Sivelestat

## Abstract

Neutrophil elastase (NE) is a proteolytic enzyme released extracellular during the formation of neutrophil extracellular traps (NETs) through degranulation. In addition to participating in the body's inflammatory response, NE also plays an important role in cancer. It can promote tumor proliferation, migration, and invasion, induce epithelial-mesenchymal transition (EMT), and change the tumor microenvironment (TME) to promote tumor progression. Concurrently, NE promotes systemic treatment resistance by inducing EMT. However, it can also selectively kill cancer cells and attenuate tumor development. Sivelestat is a specific NE inhibitor that can be used in the perioperative period of esophageal cancer patients to reduce the incidence of postoperative complications after esophagectomy. In addition, the combination of sivelestat and trastuzumab can enhance the efficacy of human epidermal growth factor receptor 2(HER 2) positive breast cancer patients. Meanwhile, targeting the human antibody domains and fragments of NE is also a new way to treat cancer and inflammation-related diseases. This review provides valuable insights into the role of NE in cancer treatment. Additionally, we discuss the challenges associated with the clinical application of sivelestat. By shedding light on the promising potential of NE, this review contributes to the advancement of cancer treatment strategies.

## Introduction

Neutrophil elastase (NE) is a serine protease primarily expressed in the primary granules of neutrophils. It is released into the extracellular space through degranulation during the formation of neutrophil extracellular traps (NETs) [1]. NE is involved in various physiological and pathological processes. Besides its role in the body’s inflammatory response [2, 3], NE also promotes the development of cancers, including breast cancer, lung cancer, prostate cancer, and colon cancer [4–8]. Sivelestat is a low molecular NE-specific inhibitor that can competitively inhibit the activity of NE without affecting other proteases [9, 10]. The drug has been approved for the treatment of acute lung injury (ALI) and acute respiratory distress syndrome (ARDS) in Japan and South Korea [11], as well as for the treatment of COVID-19 in China [12]. In February 2022, the Chinese Expert Consensus on Expert Guidance for the Clinical Application of Sivelestat was released [13]. Apart from cardiorespiratory diseases, sivelestat has also been suggested for the treatment of other inflammatory diseases and cancers [4, 14]. However, research on the use of sivelestat in cancer is still limited, and further research is needed to support its clinical application.

This review discusses the role of NE in the body’s inflammatory response and its mechanisms in promoting primary tumor growth, organ metastasis, and cancer treatment resistance. It also elaborates on the clinical application of sivelestat in cancer treatment and briefly explores the prospect of NE as a new target for cancer treatment.

## NE and its role in inflammatory response

### Molecular structure of NE

Neutrophil elastase, also known as serine elastase, leukocyte elastase, polymorphonuclear leukocytes(PMNs) elastase, and granulocyte elastase. NE is a proteolytic enzyme with a relative molecular mass of 29KDa encoded by the ELANE gene and belongs to the chymotrypsin family of serine proteases. In 1986, Bode W and others determined the molecular structure of NE through X-ray diffraction, that is, NE is a polypeptide chain consisting of 218 amino acid residues starting with Ile and ending with Gln [15]. Later, Macdonald, Koizumi, and Hansen further reported on the molecular structure of NE and found that NE contains a folding region. This folding region is composed of two barrel-like structures, and each barrel-like structure is composed of six anti-parallel structures. It is composed of β-sheets, and there are four disulfide bonds between the six β-sheets to maintain its stable structure [16–18].

### Physiological functions of NE

The main physiological function of NE is host defense, which involves the degradation of foreign microorganisms or organic molecules engulfed by neutrophils. Meanwhile, NE can degrade various extracellular matrix (ECM) proteins, such as collagen and elastin, thereby promoting neutrophil migration and tissue repair [1, 19]. In addition, it can activate and regulate a variety of cytokines and chemical mediators, such as tumor necrosis factor and interleukin, and participate in immune response and inflammation regulation [20, 21]. Under physiological conditions, the majority of NE is limited within cells or expressed on the surface of initially activated cells and will not digest normal proteins in the body. However, when PMNs are overactivated, a large amount of NE can also be released outside the cells and damage normal tissues [22].

### NE participates in the body’s inflammatory response

In 1994, Döring G and others found that NE was closely related to inflammatory response, which was not only limited to local inflammatory response but can also affect the immune status of the whole body [23]. For instance, several studies have demonstrated the presence of NE in the airways of patients with diverse lung diseases, with its expression level being correlated with pneumonia. Simultaneously, NE directly activates inflammation by increasing the expression and release of cytokines and indirectly activates inflammation by triggering NETs and exosome release, thereby amplifying protease activity and inflammation in the airways. Furthermore, it is also involved in the occurrence and development of diseases such as rheumatoid arthritis, inflammatory bowel disease, and pulmonary fibrosis [24].

NETs kill pathogenic bacteria through NE. NETs are produced by activated neutrophils and exert antimicrobial activity at low concentrations. Exposure to cytokines, chemokines, or bacterial products can lead to the accumulation of NE and anti-infectious factors like microbicidal peptides and ROS [25]. However, overactivated NE can cause the degradation of ECM and the production of oxygen-free radicals, causing tissue inflammation and damage. Concurrently, the damaged basement membrane releases laminin fragments, which facilitate leukocyte migration and the recruitment of antioxidant factors, thereby expediting the inflammatory process [3]. Moreover, NE can impair ECM components, including the vascular endothelium and fibrin, resulting in immune cell aggregation and fibrotic responses [26]. NE can stimulate and activate various cell types, including mast cells, neutrophils, macrophages, and others. Additionally, NE can promote the synthesis and release of inflammatory mediators, such as tumor necrosis factor-α (TNF-α), leukotriene B4 (LTB4), and interleukin-8 (IL-8), thus inducing an inflammatory response [26].

NE can induce the phosphorylation of the Src kinase family, promote macrophage adhesion and cytokine production through the integrin-Src kinase pathway, and reduce the phagocytic ability of macrophages to bacteria [27]. During the activation process of neutrophils, elastase can interact with cell surface receptors. NE initiates inflammation through Toll-like receptor 4 (TLR4). Intercellular adhesion molecule-1 (ICAM-1) is an integrin receptor molecule on various somatic cell surfaces. In the inflammatory response, NE activates ICAM-1 to promote the accumulation of neutrophils and other immune cells, thereby intensifying and accelerating the inflammatory response [27]. Additionally, NE can regulate inflammatory responses through the NF-κB pathway. Sivelestat affects the IKK pathway by inhibiting IκB phosphorylation and NF-κB activation to inhibit the inflammatory response [28]. By inhibiting the activity or expression of NE, the intensity and duration of the inflammatory response can be reduced, ultimately improving the prognosis of the disease.

## NE reshapes the tumor microenvironment

The tumor microenvironment (TME) is a complex environment composed of cells, molecules, and ECM that surrounds the tumor has the potential to affect tumor cell proliferation, invasion, and metastasis, and it plays a role in various stages of tumor development while also influencing the TME. Circulating neutrophils and myeloid-derived suppressor cells (MDSCs) are related to patient survival and can secrete NE and NETs [29]. NE can activate chemokines, cytokines, and other growth factors that promote the chemotaxis of MDSCs toward the TME. Consequently, inhibiting NE activity may indirectly reduce the infiltration of MDSCs. Neutrophils can not only infiltrate tumors but also alter tumor growth and invasiveness [30–32]. For instance, tumor cells can recruit neutrophils by producing chemokines, and neutrophil infiltration in tumor cells may be associated with adverse clinical outcomes [33, 34].

Previous studies have shown that NE can promote tumor development in breast cancer, lung cancer, prostate cancer, and colorectal cancer [4–8]. In a mouse model, blocking the activity of NE can significantly inhibit the effect of neutrophils on tumor progression and metastasis [4, 6, 35]. In addition, NE also promotes tumorigenesis by inactivating tumor suppressors [14, 36–38]. During tumorigenesis, the expression of NE increases both within tumors and in circulation [39–41]. NE can enter the endosomal compartment within tumor cells, degrade insulin receptor substrate-1 (IRS-1), and directly induce tumor cell proliferation in human and mouse lung adenocarcinoma [4]. NE can also indirectly promote tumor cell invasion by directly dissolving the tumor matrix or activating protease cascade reactions and is associated with poor prognosis in non-small cell lung cancer (NSCLC) patients [42]. NE released by activated neutrophils can also mediate signaling pathways related to phosphoinositide 3-kinase (PI3K) and promote the growth and progression of tumor cells [4]. Wada Y found that NE can enhance the growth and invasion activity of esophageal cancer cells. NE cleaves and releases transforming growth factor-α (TGF-α) on the cell membrane, activates epidermal growth factor receptor (EGFR), and triggers extracellular regulated protein kinases 1 and 2 (ERK 1/2) signaling pathway [14, 43]. NE may also contribute to tumor growth and metastasis by regulating angiogenesis in the TME. For instance, NETs can stimulate endothelial cell (EC) proliferation, motility, and the formation of blood vessels [44]. In addition, NE can stimulate the release of vascular endothelial growth factor (VEGF) from the surface of tumor cells, thereby activating tumor-associated EC proliferation. In mouse models with NE-related gene knockout, angiogenic markers such as VEGF and CD31 are significantly reduced [45].

## NE can serve as a potential biomarker for cancer

Some clinical studies have shown that NE levels are elevated in various types of tumors, and these levels are associated with tumor stage, grade, and survival. In NSCLC, the elevation of NE in tumor tissue is considered an independent prognostic factor. The research results of Yamashita and others indicated that the NE concentration in T4 patients was significantly higher than that in T1, T2, and T3 patients, and patients with aortic invasion had a higher NE concentration than those without [40].In an early small sample study of Yamashita and others, 34 out of 40 lung cancer tissues had elevated NE, and stage IIIB was significantly higher than stages I, II, and IIIA. Stage IIIA was significantly higher than stage I. In addition, patients with higher NE levels had shorter survival [46]. In breast cancer, patients with stages III and IV had higher NE than those with stages I and II [47]. The disease-free survival period of patients with high NE levels was significantly shortened [48]. While patients with high NE concentrations had faster recurrence and earlier death [7]. In colorectal cancer, neutrophil infiltration and NE expression were increased in cancer tissues. At the same time, serum NE concentration in colorectal cancer patients was significantly higher than that in healthy controls. To investigate the role of NE in tumor development, an in vivo imaging system (IVIS) was used to detect NE activity in xenografts of colorectal cancer cells (HCT-15). Compared with normal mice, the amount of active NE in xenografts significantly increased. Furthermore, it was found that sivelestat can inhibit tumor growth in HCT-15 induced xenografts [6]. In brain tumors, NE was present in the tumor-infiltrating regions of glioblastoma and anaplastic astrocytoma, but not in the tumor core or low-grade astrocytoma. The higher the tumor grade, the more neutrophils infiltrate in the margin, and NE may play a role in the infiltration process of glioma [49]. These results suggest that NE may be a potential biomarker for cancer and may become a future therapeutic target.

## NE promotes the malignant progression of tumors

### NE promotes the growth and proliferation of primary tumor cells

The tumor-promoting effect of NE has been verified in lung cancer, breast cancer, and colon cancer [4–6, 45]. In patients with lung cancer and colon cancer, the NE protein and activity are significantly increased in the serum compared to healthy individuals, and these increases are associated with disease progression [6, 50]. Additionally, studies using mouse models of lung and breast cancer have shown that NE deletion led to a reduction in tumor number and size [4, 5, 45]. NE may promote tumor growth by directly increasing cancer cell proliferation or by inducing angiogenesis within the TME. It may also contribute to tumorigenesis by deactivating tumor suppressors and subsequently relieving growth inhibition.

**Fig. 1:**
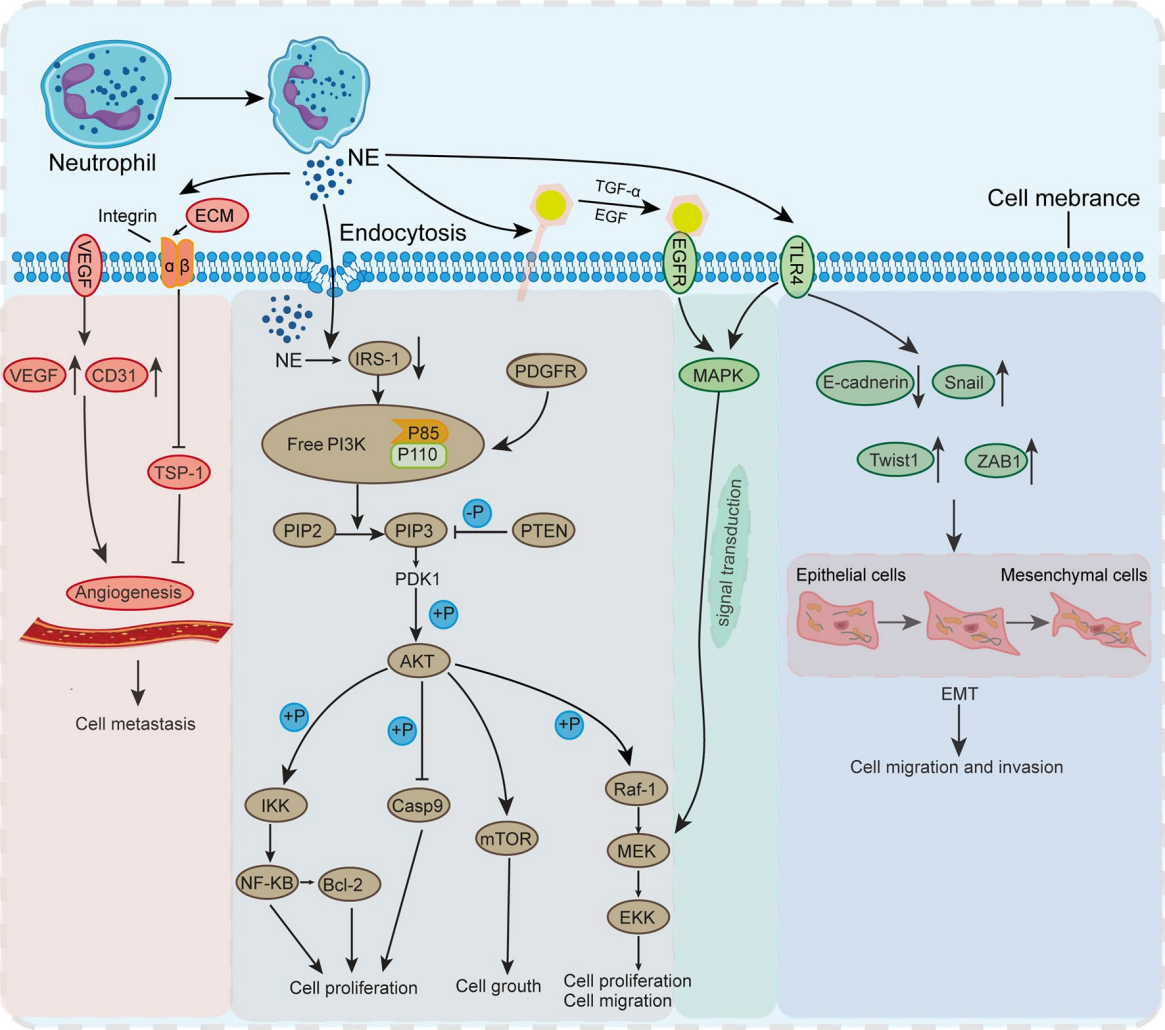
NE promotes the proliferation, migration, and invasion of tumor cells. *NE* neutrophil elastase, *IRS-1* insulin receptor substrate-1, *PI3K* phosphatidylinositol 3-kinase, *MAPK* mitogen-activated protein kinase, *AKT* protein kinase B, *IKK* inhibitor of kappa B kinase, *NF-κB* nuclear factor-kappa B, *Bcl-2* B-cell lymphoma-2, *PTEN* phosphatase and tensin homologue deleted on chromosome ten, *mTOR* mammalian target of rapamycin, *TLR4* toll-like receptor 4, *ECM* Extracellular Matrix, *TSP-1* thrombospondin-1, *EGFR* epidermal growth factor receptor, *VEGF* vascular endothelial growth factor, *EMT* epithelial-mesenchymal transitions, *PDGFR* platelet-derived growth factor receptor

Houghton AM and colleagues conducted experiments utilizing a mouse lung adenocarcinoma LSL-K-ras model to explore the impact of NE-mediated degradation of IRS-1 on lung cancer growth. They found that NE can directly enter the intracellular compartments of tumor cells through endocytosis, where IRS-1 was degraded, ultimately leading to tumor cell proliferation (Fig. 1) [4]. IRS-1, the binding partner of the p85 regulatory subunit of PI3K, undergoes degradation by the enzyme NE. As NE degrades IRS-1, the formation of a binding complex between IRS-1 and PI3K is hindered, resulting in the presence of intracellular-free PI3K. The free form of PI3K can bind to platelet-derived growth factor receptor (PDGFR), thereby inducing the tumor proliferation signaling pathway (Fig. 1). Moreover, cancer susceptibility may increase when IRS-1 is depleted by NE-mediated degradation or its biological activity is altered [43]. NE can induce the phosphorylation and activation of the p65 subunit of IKKb and NF-κB, thereby regulating the expression of B-cell lymphoma-2(Bcl-2) through NF-κB (Fig. 1) [51, 52]. Furthermore, NE regulates pro-proliferative and pro-metastatic signaling cascades in cancer cells by activating kinases, cell surface receptors, chemokines, and other growth factors. For example, NE can directly stimulate proliferative pathways and induce mitogen-activated protein kinase(MAPK) signaling through extracellular transactivation of membrane receptors such as EGFR and TLR4 (Fig. 1) [53]. In breast cancer and prostate cancer cells, NE induces ERK phosphorylation and ERK-dependent gene transcription through MAPK. Therefore, the intervention of MEK inhibitors can eliminate NE-induced proliferation [5, 8, 43]. Simultaneously, NE may cleave and release various membrane binding ligands, including EGF-like ligands, TGF-α, and platelet-derived growth factor (PDGF), to trans-activate EGFR and promote tumor cell proliferation (Fig. 1) [14, 54–56].

In addition to directly affecting tumor growth and metastasis, NE can also promote tumor progression in the lung through indirect means. NE degrades elastin as well as collagen, cadherin, fibronectin, and proteoglycans, which may lead to tumor invasion into lung tissue [57]. Moreover, some studies have revealed that curcumin can completely inhibit excessive tumor proliferation induced by NE. α1-antitrypsin is a natural inhibitor of NE. Curcumin counteracts NE-induced decrease in α1-antitrypsin by inducing the promoter activity of α1-antitrypsin and promoting its expression in lung adenocarcinoma cells. Concurrently, curcumin inhibits NE-induced proliferation by modulating the PI3K/Akt pathway [58]. p200 CUX 1 is an important tumor suppressor in hematopoietic diseases. NE can promote acute promyelocytic leukemia (APL) cell proliferation and inhibit cell differentiation by mediating proteolysis of the tumor suppressor p200 CUX 1. In addition, NE-specific inhibitors exert the same effects as interfering with NE expression, decelerating cell proliferation, inducing cell differentiation, and inhibiting proteolysis of p200 CUX 1 [59, 60].

### NE promotes tumor cell invasion and metastasis

Tumor metastasis is a complex process, starting with the degradation of the basement membrane and ECM, followed by local invasion of tumor cells into surrounding tissues [61]. Increasing evidence suggests that neutrophils contribute to cancer progression, particularly metastasis, through the release of their secretory granules [62]. NE released by activated neutrophils is a major stimulus for tumor metastasis, as NE gene deletion or pharmacological inhibition significantly reduces the possibility of tumor metastasis [63]. NE has the ability to functionally activate or modify some proteins that promote EMT, degrade ECM, and trigger angiogenesis (Fig. 1).

In the process of NETs formation, the released granulin protein NE can promote tumor cell migration and invasion by mediating ECM degradation and remodeling [64, 65]. For example, NE promotes acute myelogenous leukemia(AML) cell migration by degrading stromal cell-derived factor-1 (SDF-1) [66]. Thrombospondin-1 (TSP-1) is an ECM protein secreted in the TME. It has an inhibitory effect on NE and can also be degraded by NE. As a potent angiogenesis inhibitor, upregulation of TSP-1 is thought to inhibit tumor growth and metastasis (Fig. 1) [67]. However, in a tail vein injection model of melanoma, TSP-1 was reported to be degraded by excess released NE in the lung, thereby promoting melanoma metastatic growth in the lung. The NE/TSP-1 axis emerges as a potential anti-metastatic therapeutic target. Studies have reported that ERK serves as a key regulator of cell migration in various cancer types. NE can cleave TGF-α from the cell membrane, leading to EGFR phosphorylation and triggering the ERK signaling pathway, thus leading to the migration of colorectal cancer cells (Fig. 1). Furthermore, in a mouse model of liver metastasis from colorectal cancer, the inhibition of NE significantly reduced the formation of liver metastasis from colorectal cancer cells. Simultaneously, sivelestat can not only inhibit NE induced colorectal cancer cell migration but also suppress NETs and reduce liver metastasis [68].

Another aspect of NE related to tumor invasion and metastasis is its influence on tumor angiogenesis and the structural–functional characteristics of the new blood vessels formed. For instance, the expression of E-selectin on endothelial cells significantly increases following NE stimulation, which directly leads to the enhancement of the vascular adhesion ability of cancer cells [69]. One explanation for how NE and NETs promote metastasis and diffusion is to induce EMT, which makes cancer cells more migratory and invasive (Fig. 1). Intervening various types of cancer cells with NE can enhance the migration and invasion capabilities in vitro. For example, in ovarian cancer cells, NE downregulates the epithelial marker E-cadherin and activates β-catenin signaling [70]. In pancreatic cancer cells, NE reduces the expression of E-cadherin and keratin while inducing the expression of β-catenin-mediated mesenchymal markers ZEB1 and Twist1 [38, 71].

## NE selectively kills tumors and attenuates tumorigenesis

Cancer, as a mutational disease, demonstrates significant spatial and temporal genetic heterogeneity [72, 73]. In addition to overcoming this heterogeneity, simultaneously eliminating cancer cells and retaining non-cancer cells remains a difficult task. Additionally, developing drugs with broad efficacy and specificity across multiple cancer types is also a future challenge.

**Fig. 2:**
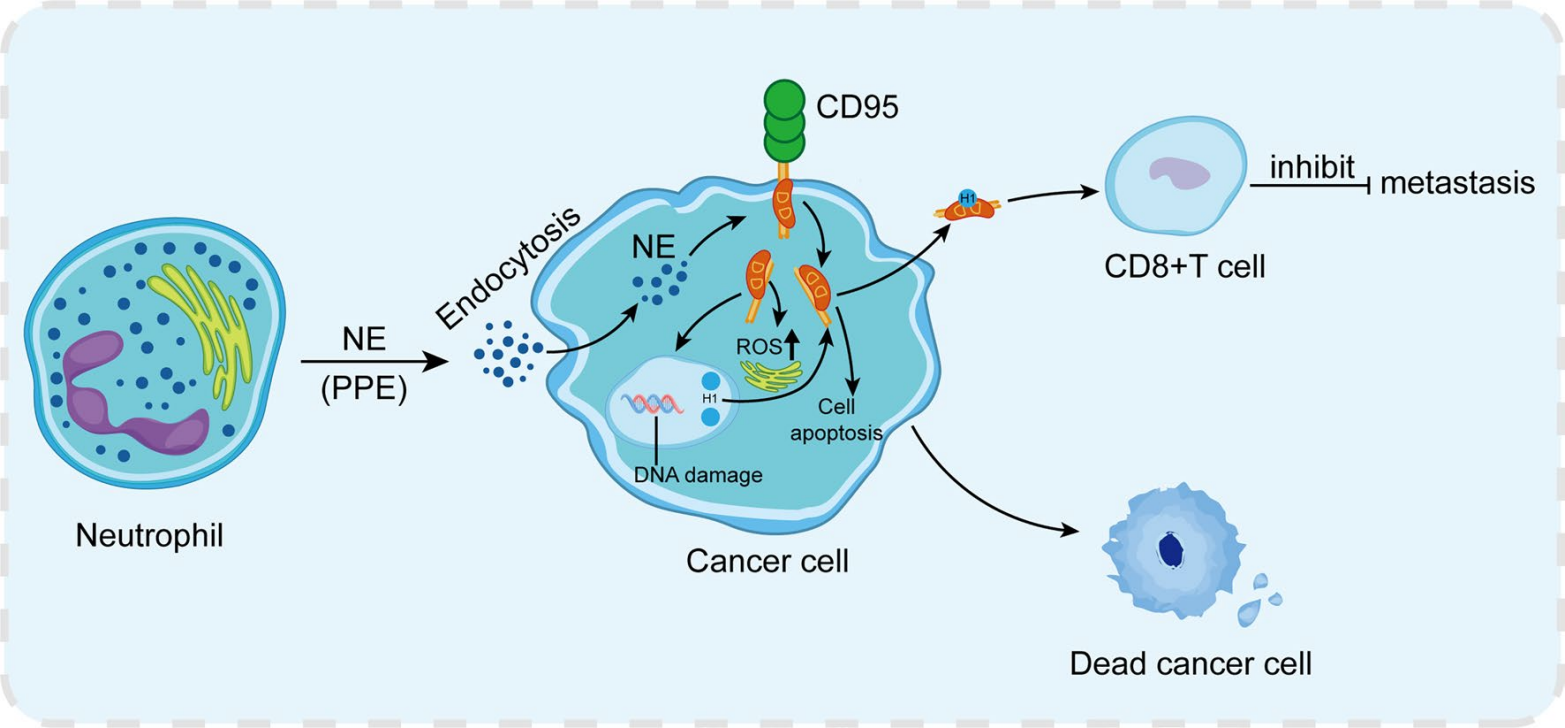
NE selectively kills cancer cells and reduces tumor development. *NE* neutrophil elastase, *PPE* porcine pancreatic elastase, *ROS* reactive oxygen species, *H1* Histone 1, *DD* death domain

Recently, a study conducted by Cui and others provided us with a new anti-cancer strategy. They found that the release of catalytically active NE from neutrophils can effectively kill many cancer cell types while having little effect on normal cells [2]. NE leaves neutrophils and enters cancer cells through receptor-mediated endocytosis. In cancer cells, it releases intracellular death domains (DD) through proteolytic cleavage of CD95 (Fas), inducing DNA damage and ROS release, ultimately leading to cell apoptosis (Fig. 2) [74]. At the same time, causing the extrusion of H1 histone subtype from the nucleus, the interaction of NE with histone H1 subtype can selectively eradicate cancer cells (Fig. 2) [75, 76]. In addition, the interaction of NE with histone H1 subtype may also produce CD8 + T cell-mediated effects in vitro to prevent distant metastasis (Fig. 2). The study also found that porcine pancreatic elastase (PPE), a NE homolog, has better systemic activity in vivo because it is resistant to serine protease inhibitors present in the tumor microenvironment. PPE can cleave CD95 and selectively kill tumor cells, similar to the effect of NE. In addition, PPE can better reduce tumor volume after in vivo injection while maintaining CD8 + T cell-dependent long-range effects [74]. In summary, NE can kill multiple types of cancer cells and has relatively low toxicity to non-cancer cells, making it a potential choice for a wide range of anti-cancer treatments.

However, the killing effect of NE on cancer has certain selectivity and specificity. First, higher levels of CD95 are observed in cancer cells. Meanwhile, the expression of histone H1 subtype is usually higher in malignant tumor cells than in non-cancer cells. Furthermore, there is an interaction between CD95-DD and histone H1 subtypes in cancer cells but not in non-cancer cells [77]. In summary, the histone H1 subtype plays a crucial role in the selective killing of cancer cells by NE. However, this anti-cancer pathway of NE may need to be experimentally verified in more cell types.

## NE promotes cancer treatment resistance

**Fig. 3:**
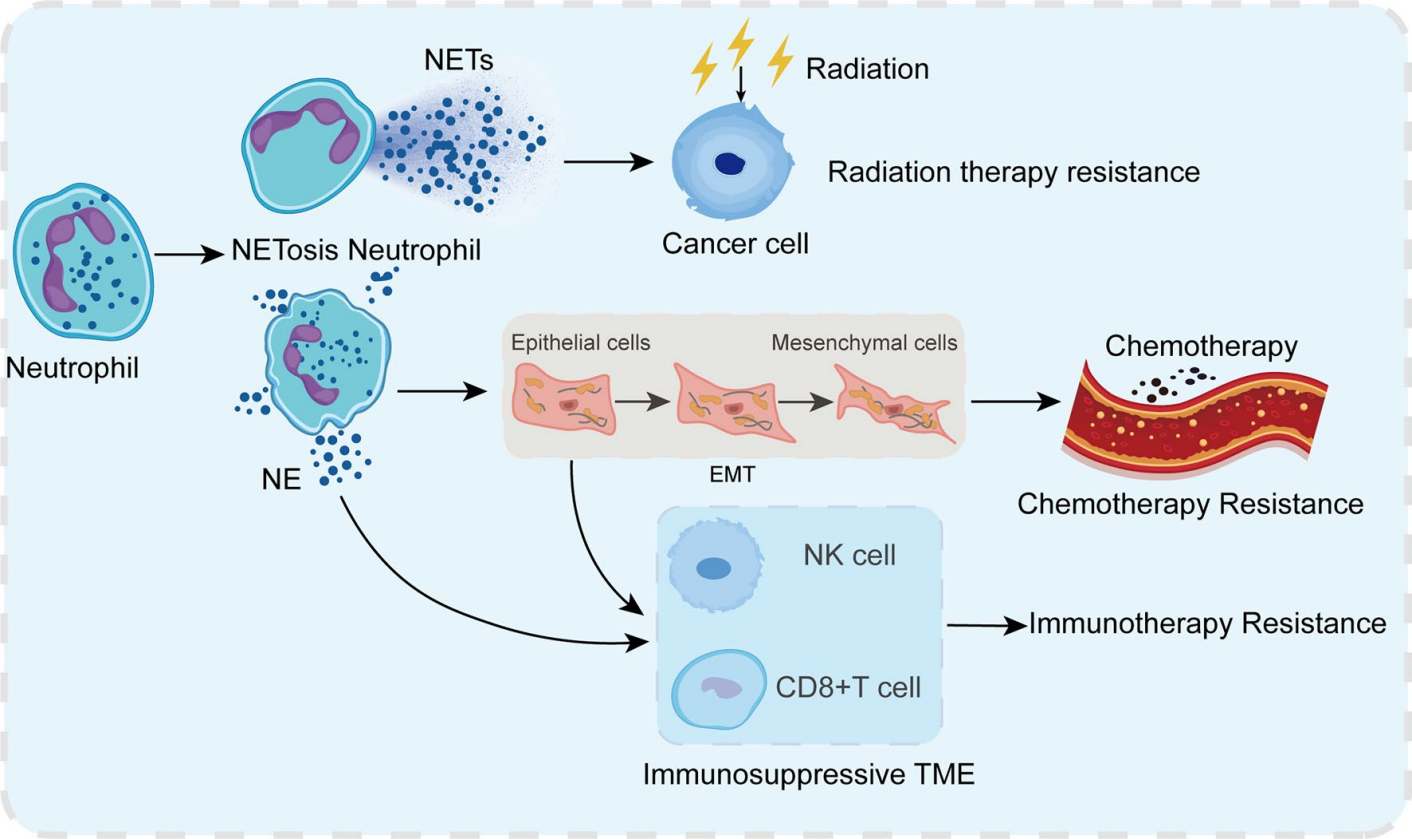
Mechanisms of NE involvement in resistance to systemic and local cancer treatment. *NE* neutrophil elastase, *NETs* neutrophil extracellular traps, *EMT* epithelial-mesenchymal transitions

Although the impact of NETs in chemotherapy, immunology, and radiotherapy has been reported, further studying the function of NETs components in drug resistance may become a new therapeutic strategy [78, 79]. Although NETs contain many components, only NE, matrix metallopeptidase 9(MMP-9) and cathepsin D(CG) have been studied in terms of tumor drug resistance [80]. The pro-tumorigenic properties of NE increase its impact on treatment response [32, 80]. Studies have shown that NE promotes systemic treatment resistance by inducing EMT (Fig. 3) [81–83]. EMT is a currently known hallmark of cancer, where cells transform into mesenchymal phenotypes with stronger migration and anti-apoptotic abilities [84–87]. The latest evidence suggests that EMT contributes to resistance to chemotherapy in multiple cancer types and may serve as a potential target for overcoming chemotherapy resistance. EMT has also been shown to be helpful in immunosuppression and resistance to immunotherapy. Although various immune cells such as CD8 + T cells and NK cells have strong anti-tumor functions, tumor cells often form mechanisms to evade immune surveillance and produce an immunosuppressive TME (Fig. 3) [83].

Immunotherapy based on blocking immunosuppressive checkpoints has achieved great success in the treatment of many tumor types. However, many tumor types are still ineffective in immunotherapy and develop therapeutic resistance. The activation of EMT may be a key process that affects the function of immune cells in the TME, participating in immune suppression and tolerance. Nevertheless, there is evidence to support that neutrophil infiltration in TME is a driving factor for NE-induced EMT [29, 32]. Considering the latest evidence supporting the role of NETs in EMT, the association between NE and EMT and the associated treatment resistance have aroused the interest of researchers. Studies have reported that NETs enhance the migration ability of cancer cells and upregulate various EMT markers [81, 82]. This effect can be eliminated by DNase-1 treatment, suggesting that NETs may play a functional role in promoting EMT through NE activity [81, 82]. Targeting the NETs-dependent, NE-mediated EMT resistance pathway can restore treatment sensitivity, but this hypothesis still needs further exploration.

## NE may be a potential target for future cancer treatment

### Application of the sivelestat in the perioperative period of patients with esophageal cancer

Esophagectomy for esophageal cancer is one of the most invasive procedures in gastrointestinal tract surgery. Pneumonia, acute lung injury (ALI) and acute respiratory distress syndrome (ARDS) are common complications after esophagectomy [88]. Severe stress (such as major surgery) can induce an excessive inflammatory response and produce a large amount of inflammatory cytokines, promoting neutrophil activation and the expression of adhesion molecules on vascular endothelial cells. The overproduction of such cytokines can trigger organ dysfunction, such as ARDS, ALI, and systemic inflammatory response syndrome (SIRS) [89, 90]. High pulmonary vascular permeability is one of the causes of early hypoxemia in the disease [91]. Neutrophils and NE are considered to play a key role in endothelial injury and increased vascular permeability in ALI [92, 93]. NE is the most effective protease that stimulates airway secretion, accelerates airway inflammation, and damages airway mucosal tissue.

As a specific inhibitor of NE, sivelestat can competitively inhibit the activity of human NE [10, 94]. A phase III trial in Japan showed that sivelestat is effective in patients with SIRS-related ALI [9, 95]. Postoperative administration of sivelestat may inhibit the severe inflammatory response after esophagectomy, increase oxygenation, reduce the duration of mechanical ventilation, and shorten the length of ICU stay [95, 96]. In addition, serum inflammatory cytokines, such as IL-1b and NE, can be inhibited by administration of sivelestat [95].

###  The combination of sivelestat and trastuzumab can enhance the efficacy of HER 2 positive breast cancer patients

NE in breast cancer cells initiates the tumor progression mechanism through TGF-α, and sivelestat can significantly inhibit this mechanism. Among them, NE cleaves EGF or TGF-α from the cell surface, inducing the activation of signal transduction in an autocrine manner, thus promoting tumor progression [97]. Breast cancer with positive expression of human epithelial HER2 has been detected in 25–30% of breast cancer patients and has a high malignant potential, resulting in a poor prognosis [98]. Trastuzumab, an anti-HER2 monoclonal antibody, can lead to downregulation of HER2, which can improve the prognosis of patients with HER2-positive breast cancer. TGF-α has been found to not only inhibit HER 2 downregulation by disrupting endocytosis and lysosomal function, but also recruit HER 2 to the cell surface [99]. Furthermore, if TGF-α expression is higher in serum or tumor tissue, the prognosis of HER 2-treated patients is significantly worse [100]. TGF-α not only acts as a ligand to promote cell proliferation, but also enhances resistance to trastuzumab by inhibiting HER 2 downregulation. Furthermore, the combination of trastuzumab and sivelestat inhibited cell proliferation better than either drug alone and did not cause impairment of TGF-α-induced downregulation of HER2. In addition, studies have found that NE can increase the phosphorylation of EGFR, HER 2, and ERK1/2 in breast cancer cells and promote cell growth. Sivelestat can block the division of growth factors induced by NE from the cell surface, and reduce the effect of NE on cell growth through cell signaling pathways and phosphorylation of EGFR and HER2 [101].

### Sivelestat inhibits the growth of gastric cancer cells by inhibiting the release of TGF-α

Under physiologically disturbed conditions, such as the presence of tumors, surgical stress, or inflammation, the balance between elastase and antiprotease is disrupted, and predominantly elastinolytic activity leads to ECM destruction. It was reported that NE can cleave TGF-α precursor located on the cell surface, resulting in the release of mature TGF-α in fibroblasts and human airway epithelial cells. The release of TGF-α activates the EGFR signaling cascade [102]. EGF, TGF-α, and their receptor EGFR play important roles in the occurrence, development, and metastasis of tumors. However, the function of NE relative to TGF-α has not been elucidated in gastric cancer cells.

Wada Y and others found that NE can stimulate gastric cancer cells to release TGF-α, which induces tyrosine kinase phosphorylation of EGFR [56]. This signal is mediated by the MEK-MAPK transduction pathway to the nucleus, causing activation of G1 cyclins, thereby stimulating the cell cycle of cancer cells. However, sivelestat can significantly inhibit NE-induced TGF-α release in gastric cancer cells [56]. Blocking the release of TGF-α may inhibit the transactivation of the EGFR signaling cascade and the MEK-MAPK transduction pathway. This inhibition also inactivates the autocrine loop between TGF-α and EGFR signaling cascades, ultimately inhibiting tumor growth and progression. In addition, stimulation of EGFR leads to the production of other growth factor-receptor systems, angiogenesis-related proteins, and matrix metalloproteinases, which play an important role in the invasion and metastasis of gastric cancer cells. Molecular targeted therapy is an effective therapeutic strategy for gastric cancer, such as cetuximab as an EGFR chimeric monoclonal antibody and gefitinib as an EGFR autophosphorylation inhibitor [103, 104]. Sivelestat can also be a good targeting agent for EGFR.

### Human antibody domains and fragments targeting NE as therapeutic candidates for cancer and inflammation-related diseases

As an important regulator of the inflammatory response [27], NE can degrade all ECM proteins and activate lung epithelial cells to produce inflammatory cytokines, which in turn activates neutrophils, leading to acute lung injury or fibrosis [1, 105]. Therefore, the inhibition of NE activity can be considered as a new drug treatment strategy in cancer and inflammatory diseases. Several NE inhibitors, including sivelestat and curcumin, have been studied in mouse cancer models. Although NE inhibitors inhibit the NE activity of cancer cells such as gastric cancer and breast cancer in vitro [56, 101], their inhibitory effect on tumor growth is still weak [6, 62].

Antibody-based therapy is a very effective and promising approach to cancer treatment due to its high specificity, high affinity for the target, and strong effector capacity. In recent years, there has been increasing interest in antibody fragments and domains as therapeutic agents, including antigen-binding fragments (Fab, 50 kDa), single-chain variable fragments (scFv, 30 kDa), and heavy-chain variable domains (VH, 15 kDa) because of their small molecular size and ideal pharmacokinetics in special clinical applications [106, 107]. Furthermore, their low immunogenicity, high stability, and small size make it easier for domain antibodies to penetrate cancer tissues and block antigens within tumors.

In the study of Chu X and others, two fully human anti-NE antibodies, VH 1D1.43 and Fab 1C 10, were demonstrated and characterized [108]. Both antibodies showed good anti-aggregation properties. In addition, these two antibodies specifically bind human NE and do not bind bovine serum albumin(BSA), myeloperoxidase(MPO) or proteinase 3 (PR 3). The effect of NE on cancer cell proliferation is achieved by its entry into cancer cells and cleavage of IRS-1 to activate the PI3K/AKT proliferation signaling pathway. Therefore, the antibody therapy strategy for NE is to block cancer cell uptake or inhibit enzyme activity. The VH domain and Fab fragment exhibit high affinity and specificity for human NE. Since the epitope of Fab 1C 10 is not near the active site of the enzyme, the possible mechanism by which Fab 1C 10 inhibits NE function is that the binding of Fab 1C 10 to NE causes a conformational change in NE, causing it to lose its catalytic activity [108]. The binding site of VH-Fc 1D1.43 is located in the vicinity of the NE active site, which can directly block the entry of substrates and inhibit NE activity [108]. The effective inhibition of NE enzyme activity and blockade of NE uptake by VH 1D1.43 and Fab 1C 10, make them promising immunotherapy candidates for cancer and inflammatory diseases.

## Thought summary

In some clinical studies, NE levels are closely related to tumor stage, grade and survival. Therefore, NE may be a new potential tumor biomarker. It can promote tumor proliferation, migration, and invasion, induce EMT, reshape the tumor microenvironment, and participate in tumor progression. However, it can also selectively kill cancer cells and attenuate tumor development. Perioperative administration of sivelestat in patients with esophageal cancer can inhibit the occurrence of complications after esophagectomy. In addition, the combination of sivelestat and trastuzumab can enhance the efficacy of HER2-positive breast cancer. Meanwhile, human antibody domains and fragments targeting NE are considered therapeutic candidates for cancer and inflammation-related diseases. In the future, further studying the mechanism of NE in cancer will have great clinical significance. At the same time, the development and application of more effective and safer NE inhibitors in tumors is also promising, such as how to combine NE inhibitors with nanobiotechnology to make them work better, which is worthy of further research in the field of cancer. In summary, NE may become a new cancer biomarker and therapeutic target. By gaining a deeper understanding of the role of NE in cancer, we can hopefully develop more effective treatments to better serve cancer patients.

## Data Availability

No datasets were generated or analysed during the current study.

## Abbreviations

NE: Neutrophil elastase
NETs: Neutrophil extracellular traps
EMT: Epithelial-mesenchymal transition
TME: Tumor microenvironment
HER 2: Human epidermal growth factor receptor 2
ALI: Acute lung injury
ARDS: Acute respiratory distress syndrome
SIRS: Systemic inflammatory response syndrome
PMNs: Polymorphonuclear leukocytes
ECM: Extracellular matrix
ROS: Reactive oxygen species
TNF-α: Tumor necrosis factor-α
LTB4: Leukotriene B4
IL-8: Interleukin-8
TLR4: Toll-like receptor 4
ICAM-1: Intercellular adhesion molecule-1
MDSCs: Myeloid-derived suppressor cells
IRS-1: Insulin receptor substrate-1
PI3K: Phosphoinositide 3-kinase
TGF-α: Transforming growth factor-α
EGFR: Epidermal growth factor receptor
EC: Endothelial cell
VEGF: Vascular endothelial growth factor
PDGFR: Platelet-derived growth factor receptor
Bcl-2: B-cell lymphoma-2
MAPK: Mitogen-activated protein kinase
SDF-1: Stromal cell-derived factor-1
TSP-1: Thrombospondin-1
DD: Death domains
PPE: Pancreatic elastase
MMP9: Matrix metallopeptidase 9
CD: Cathepsin D
